# Pretreatment apoptosis in carcinoma of the cervix correlates with changes in tumour oxygenation during radiotherapy

**DOI:** 10.1054/bjoc.1999.1059

**Published:** 2000-02-21

**Authors:** M T Sheridan, C M L West, R A Cooper, I J Stratford, J P Logue, S E Davidson, R D Hunter

**Affiliations:** CRC Department of Experimental Radiation Oncology, Paterson Institute for Cancer Research; Department of Clinical Oncology, Christie Hospital NHS Trust, Manchester, M20 4BX, UK; School of Pharmacy and Pharmaceutical Sciences, The University of Manchester, Oxford Road, Manchester, M13 9PL, UK

**Keywords:** cervix carcinoma, apoptosis, hypoxia, reoxygenation, radiotherapy

## Abstract

A relationship between hypoxia and apoptosis has been identified in vitro and in experimental tumours. The aim of this study was to investigate the relationship between apoptosis, hypoxia and the change in oxygenation during radiotherapy in human squamous cell carcinoma of the cervix. Forty-two patients with locally advanced disease underwent pretreatment evaluation of tumour oxygenation using an Eppendorf computerized microneedle electrode. Twenty-two of these patients also had a second evaluation of tumour oxygenation after receiving 40–45 Gy external beam radiotherapy. Paraffin-embedded histological sections were obtained from random pretreatment biopsies for all 42 patients. Apoptotic index (AI) was quantified by morphology on TUNEL stained sections. No correlation was found between pretreatment measures of AI and either the median pO_2_(*r* = 0.12, *P* = 0.44) or percentage of values < 5 mmHg (*r* = –0.02, *P* = 0.89). A significant positive correlation was found between AI and the change in tumour oxygenation (ratio of pre:post-treatment % values < 5 mmHg) following radiotherapy (*r* = 0.61, *P* = 0.002). The lack of correlation between apoptosis and hypoxia may occur because the Eppendorf measures both acute and chronic hypoxia, and the relative ability of acute hypoxia to induce apoptosis is unknown. These results indicate that cell death via apoptosis may be a mechanism of tumour reoxygenation during radiotherapy. © 2000 Cancer Research Campaign


*British Journal of Cancer* (2000) **82**(6), 1177–1182
© 2000 Cancer Research Campaign
Article no. bjoc.1999.1059, available online at http://www.idealibrary.com on IDEAL®

### Pretreatment apoptosis in carcinoma of the cervix correlates with changes in tumour oxygenation during radiotherapy

**MT Sheridan[1], CML West[1], RA Cooper[1], IJ Stratford[3], JP Logue[3], SE Davidson[2] and RD Hunter[2]**

[1]CRC Department of Experimental Radiation Oncology, Paterson Institute for Cancer Research and [2]Department of Clinical Oncology, Christie Hospital NHS Trust, Manchester M20 4BX, UK; [3]School of Pharmacy and Pharmaceutical Sciences, The University of Manchester, Oxford Road, Manchester M13 9PL, UK

**Summary** A relationship between hypoxia and apoptosis has been identified in vitro and in experimental tumours. The aim of this study was to investigate the relationship between apoptosis, hypoxia and the change in oxygenation during radiotherapy in human squamous cell carcinoma of the cervix. Forty-two patients with locally advanced disease underwent pretreatment evaluation of tumour oxygenation using an Eppendorf computerized microneedle electrode. Twenty-two of these patients also had a second evaluation of tumour oxygenation after receiving 40–45 Gy external beam radiotherapy. Paraffin-embedded histological sections were obtained from random pretreatment biopsies for all 42 patients. Apoptotic index (AI) was quantified by morphology on TUNEL stained sections. No correlation was found between pretreatment measures of AI and either the median $pO_2$ ($r = 0.12$, $P = 0.44$) or percentage of values < 5 mmHg ($r = -0.02$, $P = 0.89$). A significant positive correlation was found between AI and the change in tumour oxygenation (ratio of pre:post-treatment % values < 5 mmHg) following radiotherapy ($r = 0.61$, $P = 0.002$). The lack of correlation between apoptosis and hypoxia may occur because the Eppendorf measures both acute and chronic hypoxia, and the relative ability of acute hypoxia to induce apoptosis is unknown. These results indicate that cell death via apoptosis may be a mechanism of tumour reoxygenation during radiotherapy. © 2000 Cancer Research Campaign

**Keywords:** cervix carcinoma; apoptosis; hypoxia; reoxygenation; radiotherapy

Hypoxia is a common feature of human tumours (Moulder and Rockwell, 1984) and is increasingly recognized to have an important role in determining not only response to therapy (Höckel et al, 1996; Fyles et al, 1998*a*) but also tumour progression (Brown and Giaccia, 1998). Hypoxia provides a physiological pressure on tumour cells and has been shown to induce apoptosis via p53, in those cells with intact apoptotic machinery (Graeber et al, 1994, 1996; Kim et al, 1997). Apoptosis has also been found to co-localize with hypoxic regions in p53-positive mouse tumours (Graeber et al, 1996). However, hypoxia influences tumours in paradoxically opposing ways: it is lethal to cells but prolonged exposure can also have a protective effect, via the selection of cells with reduced apoptotic ability (Graeber et al, 1996). The latter can lead to therapy-resistant hypoxic areas that can act as a focus for tumour regrowth.

Changes in tumour oxygenation during radiotherapy were initially demonstrated by Badib and Webster (1969) in a mixture of carcinomas and lymphomas and have since been demonstrated in clinical studies of the cervix and head and neck (Fyles et al, 1998*b*; Lartigau et al, 1998; Stadler et al, 1998). Although pretreatment hypoxia has been shown to be a negative prognostic indicator (Höckel et al, 1993; Fyles et al, 1998*a*), the therapeutic significance of changes in oxygenation post-treatment, in terms of patient survival, remains unclear. A study by Fyles et al (1998*b*) measured oxygenation, with the Eppendorf $pO_2$ histograph, pretreatment and at the end of external beam radiotherapy, in 43 patients with cervical carcinoma. The second measure of oxygenation was not predictive of patient survival. Also, there was no difference in the disease-free survival of patients that demonstrated an improvement in oxygenation versus those that showed a decrease in oxygenation after external beam radiotherapy. The mechanisms that facilitate the changes in oxygenation are unknown, although tumour shrinkage, reduced interstitial pressure and decreased oxygen metabolism have been suggested. An understanding of the mechanisms might lead to methods for controlling/manipulating tumour hypoxia and changes in oxygenation, in an attempt to enhance tumour curability.

Although the relationship between hypoxia and apoptosis has been investigated using human tumour cell lines in vitro and animal models, no work has been carried out in human tumours. It has been suggested that clinically hypoxia, a negative prognostic indicator, is more of a problem in squamous cell carcinoma (SCC) than adenocarcinoma (Overgaard and Horsman, 1997). Apoptosis has also been shown to be a negative prognostic indicator in SCC of the cervix (Levine et al, 1995). This led to the hypothesis that pretreatment apoptosis may reflect the level of tumour hypoxia in SCC (Sheridan et al, 1999). Therefore, the aims of this study were to examine the relationships between apoptosis, hypoxia and changes in the level of oxygenation post 40–45 Gy external beam radiotherapy, in patients with SCC of the cervix.

*Received 18 June 1999*
*Revised 12 October 1999*
*Accepted 12 October 1999*

*Correspondence to:* CML West

### MATERIALS AND METHODS

### Patients

Between May 1996 and July 1997, 42 patients with locally advanced SCC of the cervix underwent pretreatment evaluation of tumour oxygenation using a $pO_2$ histograph (Model KIMOC-6650, Eppendorf, Hamburg, Germany). Twenty-two of these patients also underwent a second evaluation of tumour oxygenation, following external beam radiation to a dose of 40–45 Gy. Patients were eligible if they had tumours greater than 2 cm in diameter and were to undergo routine examination under anaesthetic as part of the staging process. Tumour size was assessed clinically, radiologically (MRI) or both. Tumour shrinkage was calculated using the clinically assessed pre- and post-treatment tumour size. Patients were staged according to the FIGO system. The study had full ethical approval and all patients gave prior informed consent. Patients were treated with radical radiotherapy as described elsewhere (Levine et al, 1995). There were three patients with stage Ib, 15 with stage IIb, 23 with stage IIIb and 1 with stage IVb disease. All patients had SCC. The mean age of the patients was 58 years (range 28–79 years).

### Measurement of tumour oxygenation

Tumour oxygenation measurements were performed using a sterile polarographic needle electrode (Eppendorf), prior to treatment in patients with primary tumours > 2 cm in diameter and post 40–45 Gy radiotherapy, in patients with clinically residual disease > 1 cm in diameter. The first measurement was taken a median of 9 days prior to the start of external beam radiotherapy (range 1–18 days) and the second, a median of 7 days post-treatment (range 4 h to 13 days), at the time of the brachytherapy insertion. Patients were anaesthetized with propofol infusion. Technical details of the Eppendorf have been described elsewhere (Vaupel et al, 1991). Measurement tracks were performed at the 12 and 6 o'clock positions as described previously (Cooper et al, 1999). The median number of tumour tracks evaluated was 4 (range 2–7) and the median number of measurements recorded was 184 (range 70–255) per patient. The median $pO_2$ and hypoxic fraction were calculated. For the purpose of this study, the 'hypoxic fraction' is defined as the percentage of values less than 5 mmHg (% values < 5 mmHg). The latter was selected prior to analysis, as it is the most commonly used parameter.

### Measurement of apoptosis

Apoptosis was measured in coded paraffin-embedded histological sections of tumour biopsy material, which was taken from random areas of the tumour, at the time of the first oxygenation measurement. The TUNEL assay was carried out on tumour sections using an Apoptag kit (Oncor, Gaithersburg, MD, USA), according to the manufacturer's instructions. Apoptosis was quantified as TUNEL-positive cells and cells with apoptotic morphology (even if TUNEL-negative). This method of quantifying apoptosis in histological sections has been shown to be reliable and specific (Sheridan et al, 1999).

### TUNEL assay

Tissue sections were deparaffinized using xylene, followed by rehydration in ethanol–water baths of descending ethanol concentrations. Nuclei of tissue sections were stripped of proteins by incubation with 20 μg $ml^{-1}$ proteinase K (Sigma) for 15 min at room temperature, after which the slides were washed in four changes of distilled water for 2 min each wash. Endogenous peroxidase activity was quenched by covering the sections with 3% hydrogen peroxide for 5 min at room temperature. The sections were then rinsed twice with phosphate-buffered saline (PBS), pH 7.4, for 5 min each time. Equilibration buffer was applied directly onto the slide and incubated at room temperature for 10–15 s, after which the excess liquid was removed. Working strength terminal deoxynucleotidyl transferase (TdT) was added directly to the sections and incubated in a humidified chamber at 37°C for 60 min. The reaction was terminated by transferring the slides to a Stop/Wash buffer for 10 min at room temperature. The slides were washed in three changes of PBS for 5 min each wash. Anti-digoxigenin-peroxidase was added directly to the slides, which were then incubated in a humidified chamber for 30 min at room temperature. Colour development was carried out using a 3,3′–Diaminobenzidine (DAB) substrate working solution. Sections were counterstained in methyl green for 4 min at room temperature.

### Quantification of AI

A cell was considered to have an apoptotic morphology if any three of the following morphological features were evident: cell shrinkage, loss of contact with neighbouring cells, chromatin condensation, margination of the chromatin, blebbing. Apoptosis affects scattered single cells not groups of adjoining cells, as is the case with necrosis (Kerr et al, 1972). Since the TUNEL assay also identifies necrotic cells (Grasl-Kraupp et al, 1995), those areas of a section which were populated with > 10 adjacent TUNEL or morphologically positive cells were avoided, as these were considered to correspond with necrotic areas. A total of 2000 cells per section were scored and the AI expressed as the percentage of cells with an apoptotic morphology and/or TUNEL positivity. The same observer evaluated reproducibility of scoring AI by making two independent scores (separated by a week), on the first ten tumour sections.

### Measurement of proliferation

The mitotic index (MI) was evaluated in each TUNEL-stained tumour section. A total of 2000 cells per section were scored and the MI expressed as the percentage of cells displaying a mitotic figure.

### Statistical analysis

The relationships between the parameters were assessed using Spearman's non-parametric correlation coefficient ($r$). All tests of statistical significance were two-sided.

### RESULTS

AI and MI was quantified in formalin-fixed, paraffin-embedded histological sections for all 42 patients. The median AI was 3.45% (range 1.10–8.30%, Table 1), while the median MI was 0.68% (range 0–2.25%). No correlation was found between the MI and AI ($r$ = 0.03, $P$ = 0.83), MI and measures of oxygenation (hypoxic

© 2000 Cancer Research Campaign

**Table 1** Apoptosis (AI) and oxygenation ($pO_2$ mmHg) measurements obtained from 42 SCC cervix patients. A second post-treatment oxygenation measurement was obtained for 22 patients

| Patient | Stage | Age (years) | Grade | AI | Pretreatment | | Post-treatment | | Fold decrease in hypoxic fraction[b] |
|---|---|---|---|---|---|---|---|---|---|
| | | | | | Median $pO_2$ mmHg | Hypoxic fraction[a] | Median $pO_2$ mmHg | Hypoxic fraction | |
| 1 | IIb | 28 | Poor | 3.60 | 0 | 84 | 2 | 65 | 1.29 |
| 2 | IIb | 44 | Poor | 2.00 | 2 | 60 | 6 | 46 | 1.30 |
| 3 | IIIb | 54 | Mod | 1.90 | 21 | 31 | 3 | 58 | 0.53 |
| 4 | IIIb | 49 | Poor | 5.05 | 0 | 95 | 46 | 25 | 3.8 |
| 5 | IIb | 76 | Mod | 2.30 | 1 | 87 | | | |
| 6 | IIIb | 34 | Mod | 2.75 | 6 | 50 | | | |
| 7 | IIb | 57 | Mod | 2.05 | 1 | 92 | | | |
| 8 | IIIb | 58 | Well | 4.10 | 2 | 91 | 21 | 39 | 2.33 |
| 9 | IIIb | 62 | Mod | 8.30 | 31 | 23 | 55 | 8 | 2.88 |
| 10 | IIIb | 65 | Mod | 1.50 | 8 | 30 | | | |
| 11 | IIIb | 77 | Mod | 6.25 | 1 | 72 | 11 | 23 | 3.13 |
| 12 | IIIb | 71 | Poor | 5.20 | 17 | 43 | 22 | 37 | 1.16 |
| 13 | IIIb | 75 | Poor | 4.05 | 4 | 55 | 20 | 22 | 2.50 |
| 14 | IIIb | 66 | Mod | 3.25 | 1 | 61 | 18 | 38 | 1.61 |
| 15 | IIIb | 69 | Well | 4.65 | 4 | 56 | | | |
| 16 | IVb | 41 | Poor | 4.55 | 3 | 78 | 3 | 86 | 0.91 |
| 17 | IIIb | 42 | Mod | 2.15 | 2 | 58 | 1 | 93 | 0.62 |
| 18 | IIIb | 67 | Mod | 2.79 | 33 | 19 | | | |
| 19 | IIb | 60 | Well | 1.40 | 1 | 78 | | | |
| 20 | IIb | 42 | Mod | 2.50 | 3 | 55 | 61 | 19 | 2.89 |
| 21 | IIb | 49 | Mod | 4.10 | 7 | 46 | | | |
| 22 | IIb | 77 | Poor | 2.30 | 1 | 82 | | | |
| 23 | IIIb | 73 | Mod | 2.65 | 25 | 41 | | | |
| 24 | IVb | 73 | Poor | 1.45 | 2 | 54 | | | |
| 25 | IIb | 49 | Mod | 4.40 | 2 | 60 | 21 | 24 | 2.50 |
| 26 | IIb | 77 | Mod | 5.65 | 5 | 47 | | | |
| 27 | IIIb | 54 | Mod | 4.40 | 3 | 88 | 3 | 60 | 1.47 |
| 28 | IIb | 29 | Mod | 2.60 | 22 | 30 | 58 | 22 | 1.36 |
| 29 | IIb | 79 | Mod | 4.65 | 1 | 72 | | | |
| 30 | Ib | 65 | Well | 3.65 | 5 | 49 | | | |
| 31 | IIIb | 58 | Mod | 1.65 | 14 | 31 | 1 | 79 | 0.39 |
| 32 | IIb | 60 | Mod | 1.65 | 20 | 34 | 18 | 43 | 0.79 |
| 33 | IIb | 48 | Mod | 4.40 | 45 | 29 | | | |
| 34 | IIIb | 68 | Mod | 1.10 | 1 | 76 | | | |
| 35 | Ib | 43 | Mod | 5.05 | 4 | 50 | 11 | 32 | 1.56 |
| 36 | IIIb | 54 | Mod | 3.30 | 4 | 59 | 22 | 32 | 1.84 |
| 37 | Ib | 72 | Mod | 1.15 | 19 | 1 | | | |
| 38 | IIIb | 38 | Poor | 2.05 | 0 | 79 | 3 | 70 | 1.13 |
| 39 | IIIb | 55 | Mod | 3.60 | 1 | 55 | | | |
| 40 | IIIb | 40 | Mod | 5.35 | 38 | 23 | 24 | 13 | 1.77 |
| 41 | IIb | 55 | Poor | 4.15 | 8 | 2 | | | |
| 42 | IIIb | 78 | Poor | 4.75 | 13 | 38 | | | |

AI – apoptotic index, $pO_2$ – partial pressure of oxygen measured using Eppendorf histography system. [a]Hypoxic fraction – % values < 5 mmHg. [b]Fold decrease in hypoxic fraction – ratio of pre:post-treatment hypoxic fractions.

fraction, $r = 0.07$, $P = 0.64$) and MI and the change in oxygenation during radiotherapy ($r = 0.09$, $P = 0.70$). Reproducibility of scoring apoptosis was demonstrated by the significant correlation between the two estimates of AI, scored independently on coded slides (with an interval of 1 week), by the same observer on the same ten sections ($r = 0.70$, $P = 0.03$).

### Hypoxia and apoptosis

Pretreatment oxygenation measures were obtained for 42 patients (Table 1). The median of the hypoxic fraction (% values < 5 mmHg) and the median $pO_2$ (mmHg) values were 55% (range 1–95%) and 4 mmHg (range 0–45 mmHg). No correlation was found between AI and pretreatment oxygenation expressed as either the median $pO_2$ (mmHg) or the % values < 5 mmHg (Figures 1 and 2).

### Change in post-radiotherapy tumour oxygenation and apoptosis

Of the 42 patients, 22 underwent a second post-treatment measure of oxygenation. For the remaining 20 it was not possible to make a second measure due to technical difficulties ($n = 12$) or tumour regression ($n = 8$). There was a significant difference between the mean pre- and post-treatment hypoxic fractions for the 22 patients (57% vs 42%, $P = 0.02$) and median $pO_2$ values (9 mmHg vs 20 mmHg, $P = 0.01$). A ratio of pre- to post-treatment % values < 5 mmHg, was used to calculate the fold increase in oxygenation after 40–45 Gy radiotherapy (median = 1.51, range 0.39–3.80). The ratio increased with increasing tumour oxygenation post-radiotherapy, i.e. decreasing hypoxic fraction. There was no correlation between the time of the second measure of oxygenation after completion of external beam radiotherapy and the change in

*© 2000 Cancer Research Campaign*

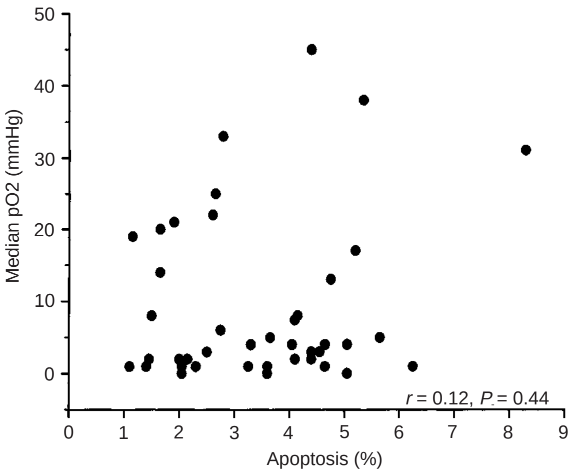

**Figure 1** The lack of relationship between AI and median $pO_2$ (mmHg) for 42 patients with SCC of the cervix. Apoptosis was measured in TUNEL-stained histological sections and quantified as TUNEL-positive cells in combination with morphology. $pO_2$ was measured in patients prior to treatment using an Eppendorf $pO_2$ histograph

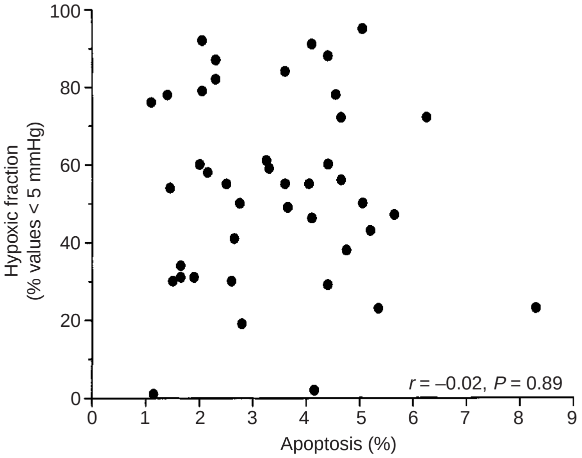

**Figure 2** The lack of relationship between AI and hypoxic fraction in 42 patients with SSC of the cervix. Apoptosis was measured in TUNEL-stained histological sections and quantified as TUNEL-positive cells in combination with morphology. The hypoxic fraction was calculated as the % $pO_2$ values less than 5 mmHg. A median of 175 measurements was recorded for each patient oxygenation ($r = 0.11$, $P = 0.59$). Figure 3 illustrates the significant positive correlation between AI and the change in oxygenation post external beam radiotherapy ($r = 0.61$, $P = 0.002$). This correlation remained, albeit with less significance, when cut-off points of 2.5 ($r = 0.48$, $P = 0.023$) and 10 mmHg ($r = 0.50$, $P = 0.019$) were used to calculate the hypoxic fraction. Finally, those tumours that had an increased level of oxygenation post-treatment, had significantly higher levels of pretreatment apoptosis than those tumours that remained unchanged or decreased in oxygenation

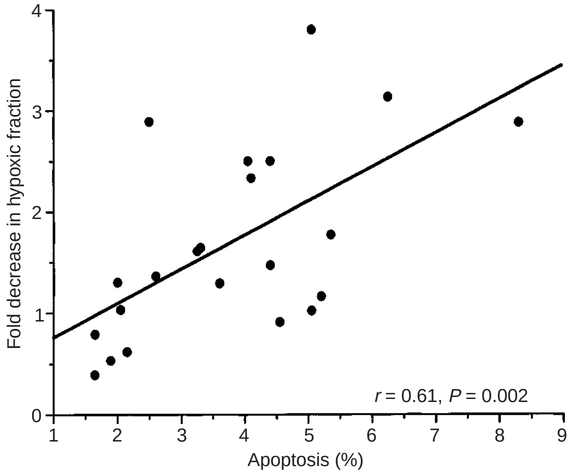

**Figure 3** The relationship between the level of apoptosis and the change in tumour oxygenation post external beam radiation, in 22 patients with SCC of the cervix. Apoptosis was measured in TUNEL-stained histological sections. The ratio of pre- to post-treatment hypoxic fraction was used to calculate the fold decrease in tumour hypoxic fraction

**Table 2** Differences in AI between tumours that showed (1) a decrease in oxygenation post 40–45 Gy, (2) an increase in oxygenation or (3) complete tumour regression

| Post-treatment | AI (%) Mean ± s.e.m. | P |
|---|---|---|
| Oxygenation ↓ ($n = 5$) | 2.38 ± 0.55 | |
| Oxygenation ↑ ($n = 16$) | 4.20 ± 0.39 | 0.02 |
| Tumour regressed ($n = 8$) | 3.40 ± 0.53 | 0.19 |

($P = 0.02$, Table 2). The correlation was lost, however if the median $pO_2$ value was used to calculate the change in oxygenation ($r = –0.16$, $P = 0.59$).

### DISCUSSION

### Hypoxia and apoptosis

Clinical studies investigating the importance of hypoxia, in relation to tumour response to therapy and prognosis, have used the Eppendorf histograph system (Lyng et al, 1997, Fyles et al, 1998*a*). In this study hypoxia was also measured using this system and expressed as median $pO_2$ mmHg and % values < 5 mmHg. The median and range of median $pO_2$ values and % values > 5 mmHg, were similar to those found by other investigators using the Eppendorf histograph system to measure oxygenation in cervical tumours (Lyng et al, 1997; Fyles et al, 1998*a*).

Tumour hypoxia can occur in two ways: diffusion-limited or chronic hypoxia and by the temporary obstruction or cessation of tumour blood flow – acute or intermittent hypoxia (Brown and Giaccia, 1998). The Eppendorf system, however, cannot differentiate between the two origins of hypoxia but measures actual oxygenation status in an area of tumour, which may contain necrotic, oxygenated or hypoxic cells. In vitro studies have examined the induction of apoptosis under chronic hypoxia (24–48 h)

© 2000 Cancer Research Campaign and found a relationship between hypoxia and apoptosis induction (Graeber et al, 1996; Kim et al, 1997). An in vivo murine tumour study also showed that apoptotic cells co-localized with hypoxic regions, detected by the hypoxia marker EF5 (Graeber et al, 1996). There are no reports, however, on the induction of apoptosis under repeated acute hypoxia. In the work reported here no relationship was seen between apoptosis and tumour hypoxia measured using the Eppendorf histograph. However, due to the limitations of the current technology, it may be difficult to establish the in vivo relationship between apoptosis and hypoxia. There may still be a relationship between apoptosis and chronic hypoxia, that could be investigated in the future using a histological marker of hypoxia alone, e.g. pimonidazole.

Another explanation for the lack of relationship between hypoxia and apoptosis in this study is the possible confounding influence of p53, or accumulated genetic alterations, on the apoptotic sensitivity of tumours to hypoxia. A study by Graeber et al (1996) investigated the relationship between hypoxic and apoptotic regions in tumours derived from E1A- and Ha-ras-transformed $p53^{+/+}$ and $p53^{-/-}$ cells and showed the importance of p53 status in influencing this relationship. Apoptotic regions were more prevalent in $p53^{+/+}$ than $p53^{-/-}$ tumours. In cervical cancer the presence of the human papillomavirus (HPV) E6 gene promotes ubiquitin-dependent degradation of wild-type p53 and results in the loss of apoptotic sensitivity to genotoxic stress such as ionizing radiation. However, it has been shown in normal epithelial cells transfected with HPV E6 and E7 that hypoxia can uncouple p53 from E6-mediated ubiquitin degradation and render the apoptotic pathway functional (Kim et al, 1997). These investigations into the relationship between hypoxia, apoptosis and HPV have been carried under controlled conditions using cell lines and murine tumours. In contrast, the study described here used human tumour biopsy material which, as well as having lost p53 function through HPV infection, may also have accumulated additional genetic alterations. This might result in loss of apoptotic sensitivity to hypoxia through a p53-independent pathway (Kim et al, 1997).

### Change in post-radiotherapy tumour oxygenation and apoptosis

A study of changes in tumour oxygenation during radiotherapy is described in detail elsewhere (Cooper et al, 1999). Overall, tumour oxygenation increased following 40–45 Gy external beam radiotherapy. For the 22 patients that had a second measure of oxygenation, the median $pO_2$ value increased from 3 mmHg to 18 mmHg and the median hypoxic fraction decreased from 59% to 38%. This change in oxygenation post-radiotherapy is similar to that seen by Lartigau et al (1998), in a study of tumour oxygenation in 14 patients with head and neck cancer. In that work, the median $pO_2$ increased after 32 Gy (2 weeks) from 12 mmHg to 26 mmHg. However, Fyles et al (1998*b*) investigated the oxygenation levels in 43 patients with cervical carcinoma, following a median dose of 50 Gy and found no significant differences between the pre- and post-treatment values. The median $pO_2$ remained at 12 mmHg and the hypoxic fraction increased post-treatment from 37% to 41%.

In this study, we have shown a significant positive relationship between the level of change in oxygenation post-radiotherapy and the AI (Figure 3). That is, those patients with a high level of pretreatment apoptosis had increased levels of post-treatment oxygenation. Although this increase in tumour oxygenation during radiotherapy measured by the Eppendorf histograph is not the same as radiobiological reoxygenation, the mechanisms involved may be similar. These have been suggested to include: reduced $O_2$ metabolism, improved circulation, tumour shrinkage, migration of surviving cells from formerly hypoxic areas into areas of more adequate oxygenation (Horsman and Overgaard, 1997).

Our results suggest that cell death by apoptosis may be facilitating reoxygenation in SCC of the cervix. To support this argument, needle track biopsies, which were taken from the region of the tumour where oxygen measurements had been performed, were examined for apoptosis after radiotherapy ($n$ = 9). Due to limited or a lack of viable tumour in these post-radiotherapy biopsies it was not possible to quantify the level of apoptosis. However, in the biopsies from patients who showed an increase in oxygenation after radiotherapy ($n$ = 6), it was observed that there was either no tumour cells ($n$ = 5) or extensive apoptosis in those that remained ($n$ = 1). Biopsies from patients whose tumours did not show an increase in oxygenation ($n$ = 3), had very low levels of apoptosis (< 1%, $n$ = 2) and viable tumour cells, while in one case no tumour was evident.

Tumour shrinkage as a result of apoptosis has previously been identified in animal models (Meyn et al, 1992, 1993; Milas et al, 1995; Rupnow et al, 1998). Tumour shrinkage would relieve pressure on blood vessels and reduce the level of intermittent hypoxia. Indeed, the study by Milas et al (1995) showed that elimination of tumour cells by apoptosis resulted in tumour reoxygenation, measured by the Eppendorf histograph, and subsequently confirmed as radiobiological reoxygenation. In the study presented here, however, no relationship was found between tumour shrinkage, measured clinically and the level of pretreatment apoptosis ($n$ = 22, $r$ = –0.32, $P$ = 0.15) or the change in oxygenation during radiotherapy ($n$ = 22, $r$ = –0.15, $P$ = 0.45). That is, those tumours that reduced in size did not necessarily display an increase in oxygenation post-treatment. However, a reduction in interstitial fluid pressure (IFP) can also lead to increased tumour oxygenation, independent of size (Milosevic et al, 1998). Therefore, it is hypothesized that the change in oxygenation observed in this study may be due to reduced IFP as a result of cell loss via apoptosis. The cell loss would relieve pressure on vessels, increasing tumour blood flow and thus oxygen delivery, without affecting tumour size. Further investigations are required to test this hypothesis.

### Clinical implications

Two studies of adenocarcinoma of the cervix have shown a correlation between high levels of pretreatment apoptosis and a good prognosis (Wheeler et al, 1995; Sheridan et al, 1999). In contrast, two studies of predominantly SCC of the cervix showed that high apoptosis predicted for a poor prognosis (Levine et al, 1995; Tsang et al, 1999). These studies used similar methods for measuring apoptosis and had comparable radiotherapy treatment regimes. We hypothesized previously that the conflicting results are due to a higher incidence of hypoxia in SCC versus adenocarcinoma (Sheridan et al, 1999), and that in SCC there may be an association between hypoxia and apoptosis. The results reported here, however, using an Eppendorf $pO_2$ histograph, showed no relationship between hypoxia and apoptosis. Nevertheless, a relationship was found between apoptosis and reoxygenation – an event traditionally thought to be a positive factor during radiotherapy. As stated above, however, apoptosis in SCC is also related to a poor

*© 2000 Cancer Research Campaign* 

prognosis. It is hypothesized, therefore, that reoxygenation and nutritional replenishment may lead to accelerated repopulation, a more aggressive tumour and a poor prognosis. A larger number of patients and longer follow-up are required to investigate the relationship between re-oxygenation and patient survival.

Finally, the results presented in this study suggest two possible clinical implications for the novel treatment of cervical carcinoma, which aims to manipulate apoptosis through novel gene therapy approaches. Firstly, in adenocarcinomas, apoptosis has been shown to relate to improved survival following radiotherapy and so a therapeutic intervention, which would enhance tumour apoptosis should be beneficial. Secondly, and in contrast, for SCC, apoptosis has been show to relate to poor survival (Levine et al, 1995) following radiotherapy, and so this would suggest that, enhancing the level of apoptosis (at least during radiotherapy) might prove detrimental to the patient.

In conclusion, no relationship was found between apoptosis and hypoxia. There was, however, a significant positive correlation between apoptosis and the change in oxygenation after radiotherapy. This indicates that cell death via apoptosis may facilitate reoxygenation in SCC of the cervix.

### ACKNOWLEDGEMENTS

Discussions with Professor J Hendry are gratefully acknowledged. This investigation was supported by the European Union and the Cancer Research Campaign, UK.

### REFERENCES

Badib AO and Webster JH (1969) Changes in tumour oxygen tension during radiation therapy. *Acta Radiol Ther Physics Biol* **8**: 247–257

Brown MJ and Giaccia AJ (1998) The unique physiology of solid tumours: opportunities (and problems) for cancer therapy. *Cancer Res* **58**: 1408–1416

Cooper RA, West CML, Logue JP, Davidson SE, Miller A, Roberts S, Stratford IJ, Honess DJ and Hunter RD (1999) Changes in oxygenation during radiotherapy in carcinoma of the cervix. *Int J Radiat Oncol Biol Phys* **45**: 119–126

Fyles AW, Milosevic M, Wong R, Kavanagh M, Pintilie M, Sun A, Chapman W, Levin W, Manchul L, Keane TJ and Hill RP (1998*a*) Oxygenation predicts radiation response and survival in patients with cervix cancer. *Radiother Oncol* **48**: 149–156

Fyles AW, Milosevic M, Pintilie M and Hill RP (1998*b*) Cervix cancer oxygenation measured following external radiation therapy. *Int J Radiat Oncol Biol Phys* **42**: 751–753

Graeber TG, Peterson JF, Tsai M, Monica K, Fornace AJ and Giaccia AJ (1994) Hypoxia induces accumulation of p53 protein, but activation of a $G_1$-phase checkpoint by low-oxygen conditions is independent of p53 status. *Mol Cell Biol* **14**: 6264–6277

Graeber TG, Osmanian C, Jacks T, Housman DE, Koch CJ, Lowe SW and Giaccia AJ (1996) Hypoxia-mediated selection of cells with diminished apoptotic potential in solid tumours. *Nature* **379**: 88–91

Grasl-Kraupp B, Ruttkay-Nedecky B, Koudelka H, Bukowska K, Bursch W and Schulte-Hermann R (1995) In situ detection of fragmented DNA (TUNEL assay) fails to discriminate among apoptosis, necrosis, and autolytic cell death: a cautionary note. *Hepatology* **21**: 1465–1468

Höckel M, Knoop C, Schlenger K, Vorndran B, Baußmann E, Mitze M, Knapstein PG and Vaupel P (1993) Intratumoral $pO_2$ predicts survival in advanced cancer of the uterine cervix. *Radiother Oncol* **26**: 45–50

Höckel M, Schlenger K, Aral B, Mitze M, Schäffer U and Vaupel P (1996) Association between tumour hypoxia and malignant progression in advanced cancer of the uterine cervix. *Cancer Res* **56**: 4509–4515

Horsman MR and Overgaard J (1997) The oxygen effect. In: *Basic Clinical Radiobiology*, Steel G (ed), pp. 132–140. Arnold: London

Kerr JFR, Wyllie AH and Currie AR (1972) Apoptosis: a basic biological phenomenon with wide ranging implications in tissue kinetics. *Br J Cancer* **26**: 239–257

Kim CY, Tsai MH, Osmanian C, Greaber TG, Lee JE, Giffard RG, DiPaolo JA, Peehl DM and Giaccia AJ (1997) Selection of human cervical epithelial cells that possess reduced apoptotic potential to low-oxygen conditions. *Cancer Res* **57**: 4200–4204

Lartigau E, Luisinchi A, Weeger P, Wibault P, Luboinski B, Eschwege F and Guichard M (1998) Variations in tumour oxygen tension ($pO_2$) during accelerated radiotherapy of head and neck carcinoma. *Eur J Cancer* **34**: 856–861

Levine EL, Renehan A, Gossiel R, Davidson SE, Roberst SA, Chadwick C, Wilks DP, Potten CS, Hendry JH, Hunter RD and West CML (1995) Apoptosis, intrinsic radiosensitivity and prediction of radiotherapy response in cervical carcinoma. *Radiother Oncol* **37**: 1–9

Lyng H, Sundfør K, Tanum G and Rofstad EK (1997) Oxygen tension in primary tumours of the uterine cervix and lymph node metastases of the head and neck. In: *Oxygen Transport to Tissue XIX*, Harrison and Deply (eds), pp. 55–60. Plenum Press: New York

Meyn RE, Milas L, Story MD, Ang KK, Tomasovic SP and Stephens C (1992) Programmed cell death in response of normal and tumour tissue to radiation. *Cancer Bull* **44**: 80–85

Meyn RE, Stephens LC, Ang KK, Hunter NR, Brock WA, Milas L and Peters LJ (1993) Heterogeneity in the development of apoptosis in irradiated murine tumours of different histologies. *Int J Radiat Biol* **64**: 583–591

Milas L, Günderoth M and Peters LJ (1995) Drug and radiation-induced apoptosis as a mechanism of reoxygenation. In: *Tumour Oxygenation*, Vaupel PW, Kelleher DK and Günderoth M (eds), pp. 165–173. Gustav Fisher Verlag: Stuttgart

Milosevic MF, Fyles AW, Wong R, Pintilie M, Kavanagh M-C, Levin W, Manchul LA, Keane TJ and Hill RP (1998) Interstitial fluid pressure in cervical carcinoma. *Cancer* **82**: 2418–2426

Moulder JE and Rockwell S (1984) Hypoxic fractions of solid tumours: experimental techniques, methods of analysis, and a survey of existing data. *Int J Radiat Oncol Biol Phys* **10**: 695–712

Overgaard J and Horsman MR (1997) Overcoming hypoxic cell radioresistance. In: Basic Clinical Radiology, GG Steel (Ed), pp 141–151. Arnold: London

Rupnow BA, Murtha AD, Alarcon RM, Giaccia AJ and Knox SJ (1998) Direct evidence that apoptosis enhances tumour responses to fractionated radiotherapy. *Cancer Res* **58**: 1779–1784

Sheridan MT, Cooper RA and West CML (1999) A high ratio of apoptosis to proliferation correlates with improved survival after radiotherapy for cervical adenocarcinoma. *Int J Radiat Oncol Biol Phys* **44**: 507–512

Stadler P, Feldman HJ, Creighton C, Kau R and Molls M (1998) Changes in tumour oxygenation during combined treatment with split-course radiotherapy and chemotherapy in patients with head and neck cancer. *Radiother Oncol* **48**: 157–164

Tsang RW, Fyles AW, Li Y, Rajaraman MM, Chapman W, Pintilie M and Wong S (1999) Tumor proliferation and apoptosis in human uterine cervix carcinoma II: correlations with clinical outcome. *Radiother Oncol* **50**: 93–101

Vaupel P, Schlenger K, Knoop C and Höckel M (1991) Oxygenation of human tumours: evaluation of tissue oxygen distribution in breast cancers by computerised $O_2$ tension measurements. *Cancer Res* **51**: 3316–3322

Wheeler JA, Stephens LC, Tornos C, Eifel PJ, Ang KK, Milas L, Allen PK and Meyn RE (1995) ASTRO research fellowship: apoptosis as a predictor of tumor response to radiation in stage IB cervical carcinoma. *Int J Radiat Oncol Biol Phys* **32**: 1487–1493

*© 2000 Cancer Research Campaign*
